# On Treatment by Suggestion without Sleep

**Published:** 1907-07-06

**Authors:** Edwin Ash

**Affiliations:** sometime Demonstrator of Physiology and House Physician at St. Mary's Hospital, W.


### The General Practitioner's Column.

[Contributions to this Column are invited, and if accepted will be paid for.]

### ON TREATMENT BY SUGGESTION WITHOUT SLEEP.

By EDWIN ASH, M.B., B.S.,Lond., M.R.C.S.,Eng. (sometime Demonstrator of Physiology and House Physician at St. Mary's Hospital, W.).

Although the therapeutic efficacy of hypnotism is now well established, a great aversion to its use is manifested by both the medical profession and the public. This is perhaps not strange when we remember that hypnotic treatment necessitates a complete surrendering of faculties to the operator with a period of unconsciousness for the patient. The objection persists in spite of all that has been written and said in explanation of hypno-therapeutics and of the harmlessness of somnambulism when controlled by an experienced agent. In consequence, when my investigations were directed from the experimental aspect of the influence of suggestion to its therapeutic application, I endeavoured to confirm the observations of numerous contemporaries that suggestive therapeutics can be successfully practised without the induction of sleep. And the results obtained have shown me that by the careful application of a certain technique Direct Suggestion will do much to relieve a large number of functional neuroses. Indeed, it is remarkable to find how pain and hyperæsthesia of special senses can be alleviated by a simple system of suggestive therapy, in the application of which *one's patients are conscious of all that is being done, and moreover retain perfect liberty of thought and action.*

The technique indicated is familiar to all students of the Nancy School, and depends on gaining the patient's confidence, and verbally repeating the necessary suggestions until the requisite curative idea has been thoroughly established in his Subconscious Mind. Everyone who is practically acquainted with the methods of suggestion knows that the secret of success lies firstly in a preliminary fixation of the patient's attention on the physician to the extinction of all other things; and secondly, in an untiring repetition of the required suggestions. The process can be aided by transferring the patient's attention to the affected part, which is best done by placing one's hand over the area in question. There is nothing mysterious in this; one is desirous of keeping the patient's thoughts on some particular symptom and directs his attention thereto by touching this or that particular region. The whole method is based on the simple psychological principles of attention and ideation.

There is no doubt that the principle of suggestion has been the paramount factor in numerous "healing systems" that have been exploited recently. Indeed its indirect application has resulted in the extraordinary cures that are constantly reported by the adherents of such systems, by the vendors of patent medicines and of wondrous electrical appliances, and occasionally by a medical man astonished at the result obtained by the use of some simple remedy. Yet the profession as a whole has refused to credit the practical importance of direct or verbal suggestion, with the result that few of its members have dared openly to acknowledge its use. Those few have been rewarded by their successful efforts in treatment as a set off to the opposition, and even ridicule, of their medical brethren.

That most able exponent of this method of treatment—Dr. Bernheim, of Nancy—contended that suggestion is always beneficial, sometimes curative. And a reference to my case-books will support his contention better than any theoretical argument

Among the first cases I treated by suggestion without sleep were two of functional enuresis nocturna. The first of these was a girl 15 years of age, who was unable to remain in service owing to the unpleasant nature of her affliction. She had 14 sittings during three months, suggestions being given that she should awaken with intent at a certain time each night. There was definite improvement after the third sitting, and complete recovery after the tenth. At no time during the treatment was the patient unconscious. The other case of this description was also relieved, and was very interesting because I first treated him in the somnambulic state without result. So I tried suggestion in the waking state with fixed attention, and obtained immediate improvement, with ultimate cure. The condition of these young people with enuresis nocturna is most distressing, and if no organic cause can be found it seems desirable, after a consideration of the above results, that they should be given the benefit of treatment by suggestion as soon as it is found that the orthodox medical remedies are doing no good.

Another class of case in which excellent results can be obtained is that which exhibits insomnia with or without accompanying neuroses. The sleeplessness can often be relieved immediately so as to break the vicious cycle of events; and an improvement in the condition of general depression and "nervousness" which usually accompanies the insomnia can then be expected. It is not only as an absolute curative agent that suggestion should find a place in the armoury of modern therapeutics, for in the method of treatment by suggestion we have a ready means of relieving distressing symptoms in diseases that may be in themselves incurable. For example, I have in actual practice effectively relieved the severe pains of tabes dorsalis by suggestion; and on another occasion stopped an abdominal pain.

The method I now use exclusively is that of fixation of attention with repetition of verbal suggestions, without any loss of consciousness or will on the part of the patients. At the same time I insist on the treatment being carried out under the most advantageous conditions, and am always very careful to build up simultaneously an impoverished system by due attention to dietetic and hæmatinic measures.

